# Effect of Bracket Slot and Archwire Dimension on Posterior Tooth Movement in Sliding Mechanics: A Three-dimensional Finite Element Analysis

**DOI:** 10.7759/cureus.5756

**Published:** 2019-09-25

**Authors:** Nausheer Ahmed, Priya Megalan, Shraddha Suryavanshi, Nishat Sidiqha, Kiran Kumar Neelakantappa

**Affiliations:** 1 Orthodontics and Dentofacial Orthopedics, Government Dental College and Research Institute, Bengaluru, IND; 2 Orthodontics and Dentofacial Orthopedics, Akshaya Dental Clinic, Bengaluru, IND; 3 Endodontics, Government Dental College and Research Institute, Bengaluru, IND

**Keywords:** arch wire, bracket, power arm, protraction, fem, miniscrews

## Abstract

Introduction

Space closure by molar protraction has always been a challenge in orthodontic treatment due to larger root surface area which requires additional anchorage. Ideally, there should be little or no tipping. However, the protraction forces, being occlusal and buccal to the centre of resistance (CR) of the tooth, cause tipping and rotations.

Aim

The aim of the study was to assess the effect of bracket slot and archwire dimensions on posterior tooth movement during space closure in sliding mechanics and evaluate the length of power arm to bring about translatory movement of teeth using three-dimensional finite element analysis.

Materials and methods

A model of the maxillary teeth was created and converted to a finite element format through a meshing software, Hypermesh. Two three-dimensional models, each with a combination of 0.017”× 0.022” archwire in 0.018” slot (model 1) and 0.019”×0.025” archwire in 0.022” slot (model 2), were generated. Power arms of different lengths were attached to the first molar. Miniscrew was inserted between the canine and first premolar.

Results

In model one, the power arm of 10-mm height provided controlled tooth movement than the one with 6 mm height, and in model two, power arms of both 6-mm and 10-mm height produced controlled tooth movement.

Conclusions

As the force was raised apically from the slot, more translation was observed. Power arm of 6-mm height can be used due to anatomic limitation of the vestibule.

## Introduction

The presence of minimal crowding or protrusion can be treated by space closure with the mesial movement of molars. Second premolars are preferred for extraction in these cases [1]. Mechanically, a tooth is a reinforced, rigid body, with its support in the surrounding tissue. When the crown is loaded with a force couple, it will rotate around a well-defined axis, the so-called centre of resistance (CR), resulting in tipping and rotation of the tooth in the direction of the pull.

Hence, for translation, the force must be directed through the CR, located at the junction of the apical and middle third for the single-rooted teeth and apical to the furcation area in multi-rooted teeth [2].

However, the molar protraction forces, being occlusal and buccal to the CR, produce moments [3]. Forces could be transferred to any height in sliding mechanics, to move the tooth in a pre-programmed direction, by altering the length of the power arm [2,4]. As molar protraction is challenging and demands additional anchorage, temporary anchorage devices (TADs) can be used for providing absolute anchorage.

The finite element method (FEM) analysis, being a contemporary research tool, is a highly precise technique in orthodontics to evaluate different loading conditions to optimize the biomechanics delivered. It is also able to overcome the disadvantages of other experimental methods, as it is accurate and non-invasive and controls the study variables and provides quantitative data [5].

The purpose of this study was to assess the effect of the bracket slot, archwire dimensions, and varying heights of power arm on posterior tooth movement during space closure by sliding mechanics using a three-dimensional FEM.

## Materials and methods

This study involved finite element analysis carried out using three-dimensional models of maxillary teeth with the aid of software.

Construction of the model

**Figure 1:**
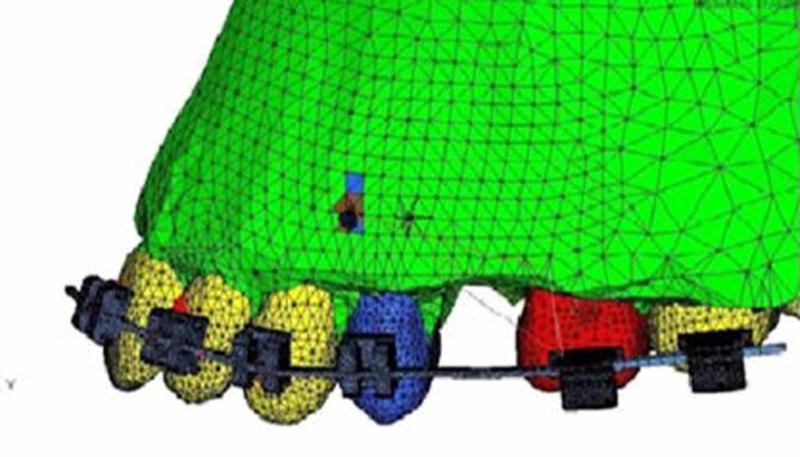
Representative model showing bracket sites and miniscrew placement

A geometric model of the maxillary base and anterior and posterior teeth was created through CT scan and converted to a three-dimensional step file format. Further, it was converted into finite element format through a meshing software, Hypermesh (Figure 1). Three-dimensional models of the periodontal ligament (PDL), alveolar bone, bracket, arch-wire, power arm, and miniscrew were constructed using the modelling and meshing software.

Two three-dimensional solid models were constructed, each with a combination of 0.017”× 0.022” stainless steel archwire in 0.018” slot (model 1) and 0.019”× 0.025” archwire in 0.022” slot (model 2). Power arms of 0.019”×0.025” stainless steel wire of different lengths were attached to the maxillary first molar. A miniscrew of 2.3-mm diameter and 8-mm length was inserted between the canine and first premolar at 8-mm height from the alveolar crest.

Model 1 (a, b, and c): 0.018” bracket slot and 0.017”× 0.022” stainless steel archwire with power arm of 2 mm, 6 mm, and 10 mm height, respectively.

Model 2 (a, b, and c): 0.022” bracket slot and 0.019” × 0.025” stainless steel archwire with power arm of 2 mm, 6 mm, and 10 mm height, respectively.

The geometric model was converted into a finite element model through ANSYS software version 11.0 (Canonsburg, Pennsylvania; USA)

Material properties and data representation

The assignment of material properties to FEM is necessary to simulate the behavior of the object studied. The different structures involved in this study include the alveolar bone, periodontal ligament, teeth, bracket, archwire, power arm, and implant. The archwire, bracket, and power arms were considered to be made of stainless steel and these structures were modelled as being homogenous and isotropic for the same reason and the implant was considered to be made of titanium alloy. The material properties assigned were Young’s modulus (or modulus of elasticity) and the Poisson’s ratio, as shown in Table 1.

**Table 1:** Material properties

Materials	Young’s Modulus (Mpa)	Poisson’s Ratio
Tooth	20,000	0.30
Periodontal ligament	0.05	0.30
Alveolar bone	2,000	0.30
Bracket/ archwire/power arm	200,000	0.30
Miniscrew	110,000	0.35
Cortical bone	13,700	0.30
Cancellous bone	1,600	0.30

Experimental conditions and force application

A protraction force of two N was applied from the miniscrew to the power arm at 2 mm, 6 mm, and 10 mm, respectively, for the mesial movement of molars on model one and model two respectively. The deformation of the archwire and the movement of maxillary posterior teeth with various heights of force application were assessed.

## Results

When a load of two N was applied from the miniscrew to the power arm at various heights, the results obtained for molar displacements and the relationship between the degree of mesiodistal tipping (crown and root displacement) of the maxillary first molar in both models are shown in Table 2.

**Table 2:** Molar displacement

Experimental model	Position of power arm (mm)	Crown displacement (µmm)	Root displacement (µmm)	Difference (µmm)	Ratio
0.017”x0.022” archwire in 0.018” slot. [model 1]					
Model 1(a)	2	8.75	1.91	6.84	0.21
Model 1(b)	6	1.22	0.57	0.65	0.46
Model 1(c)	10	0.09	0.16	0.07	1.7
0.019” x 0.025” arch wire in 0.022” slot (model 2)					
Model 2(a)	2	8.65	1.82	6.74	0.21
Model 2(b)	6	1.75	1.26	0.49	0.72
Model 2(c)	10	0.52	0.24	0.28	0.46

**Figure 2:**
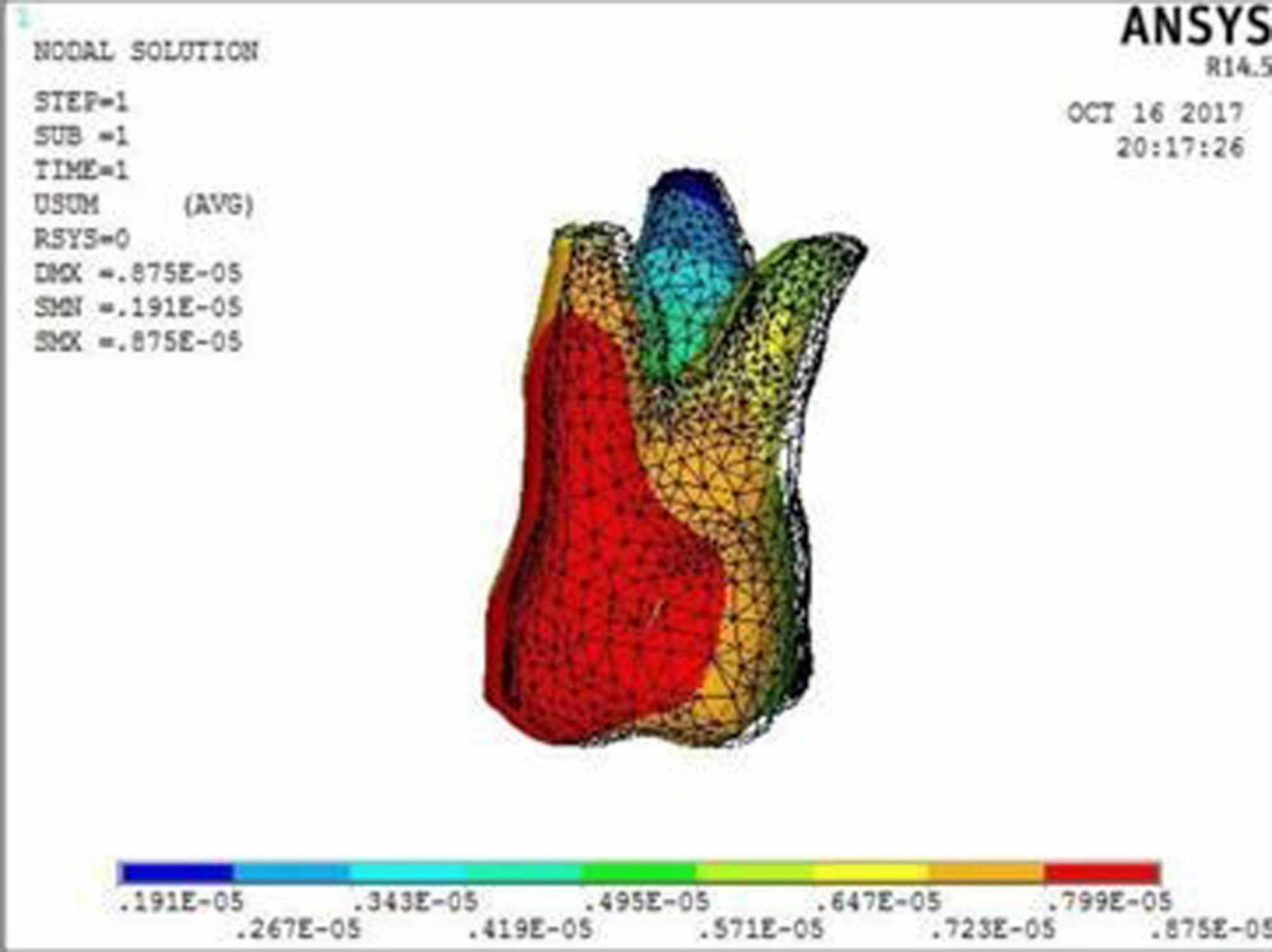
Molar displacement with 0.017” x 0.022” archwire in 0.018” slot bracket at the power arm height of 2 mm showing crown displacement of 8.75 µmm and root displacement of 1.91 µmm

**Figure 3:**
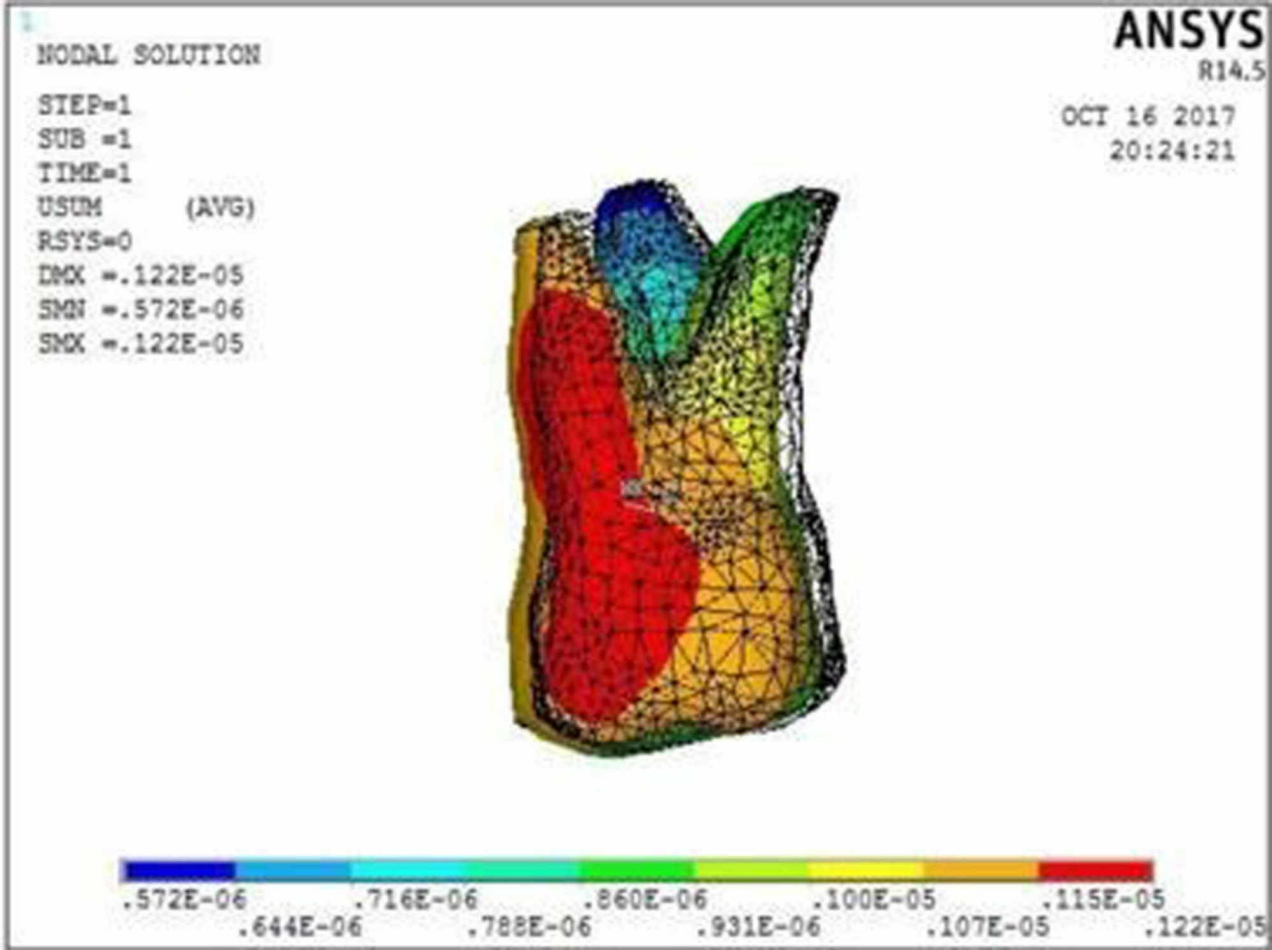
Molar displacement at the power arm height of 6 mm showing crown displacement of 1.22 µmm and root displacement of 0.57 µmm

**Figure 4:**
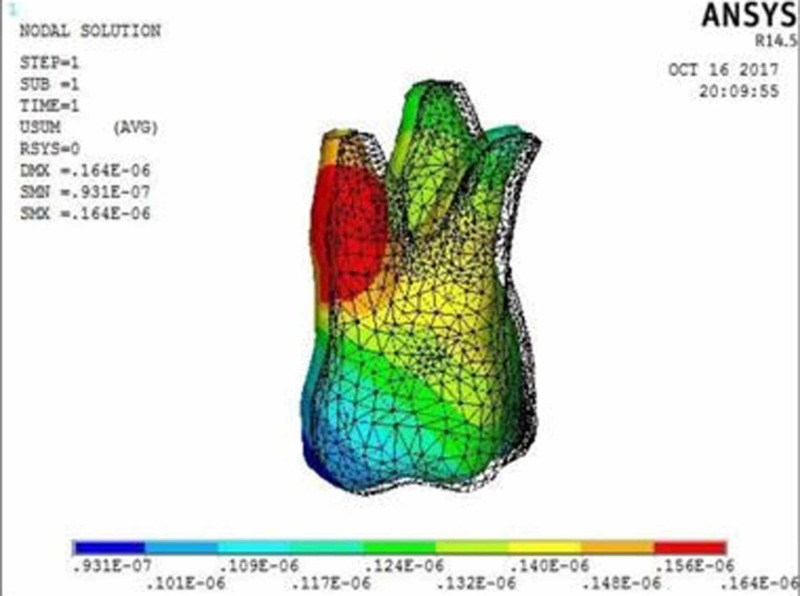
Molar displacement at the power arm height of 10 mm showing crown movement of 0.09 µmm and root movement of 0.16 µmm

The molar displacement in model 1 at the power arm height of 2, 6, and 10 mm is shown in Figures 2-4, respectively.

**Figure 5:**
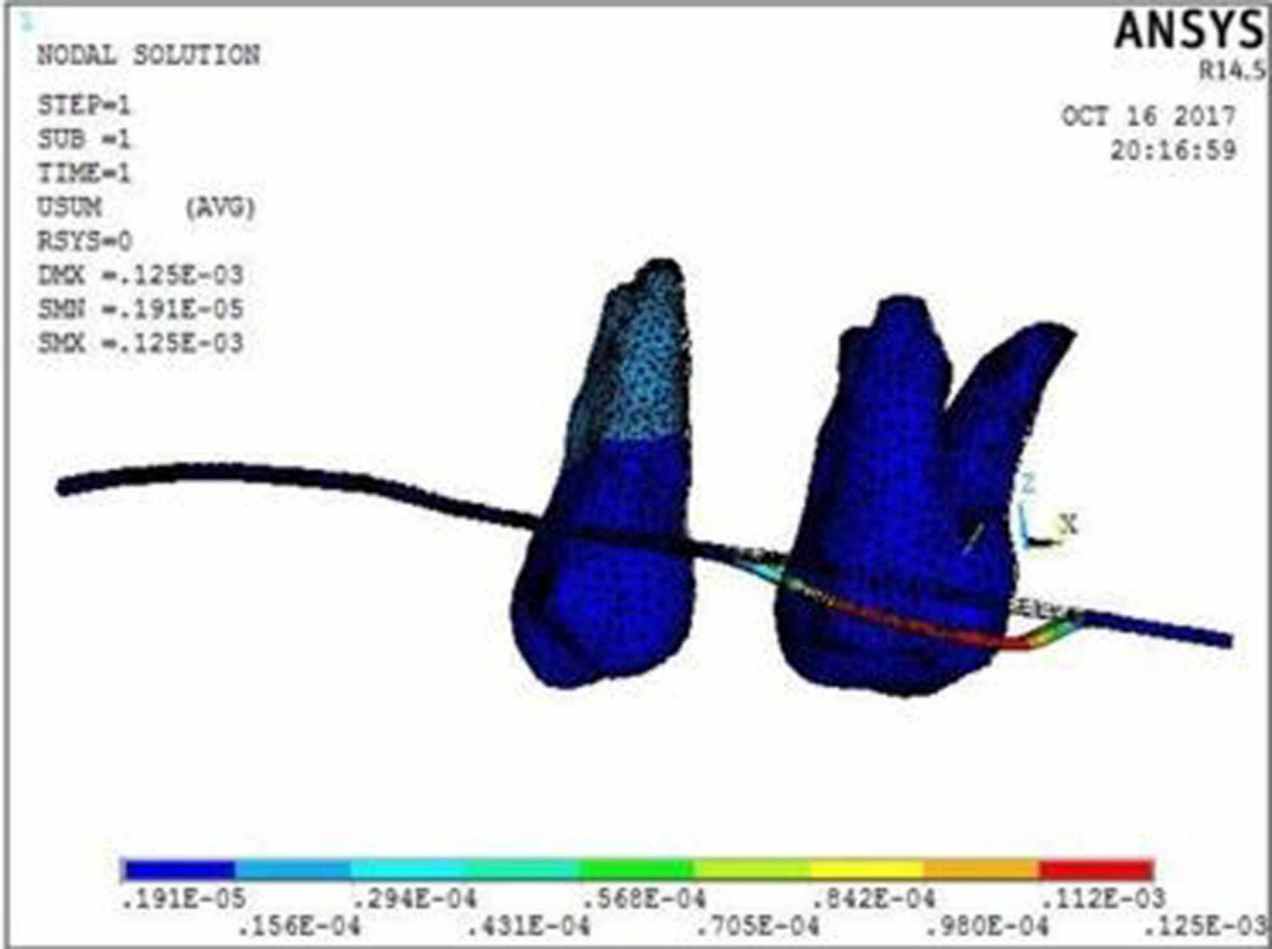
Deformation in the archwire at the power arm height of 2 mm

**Figure 6:**
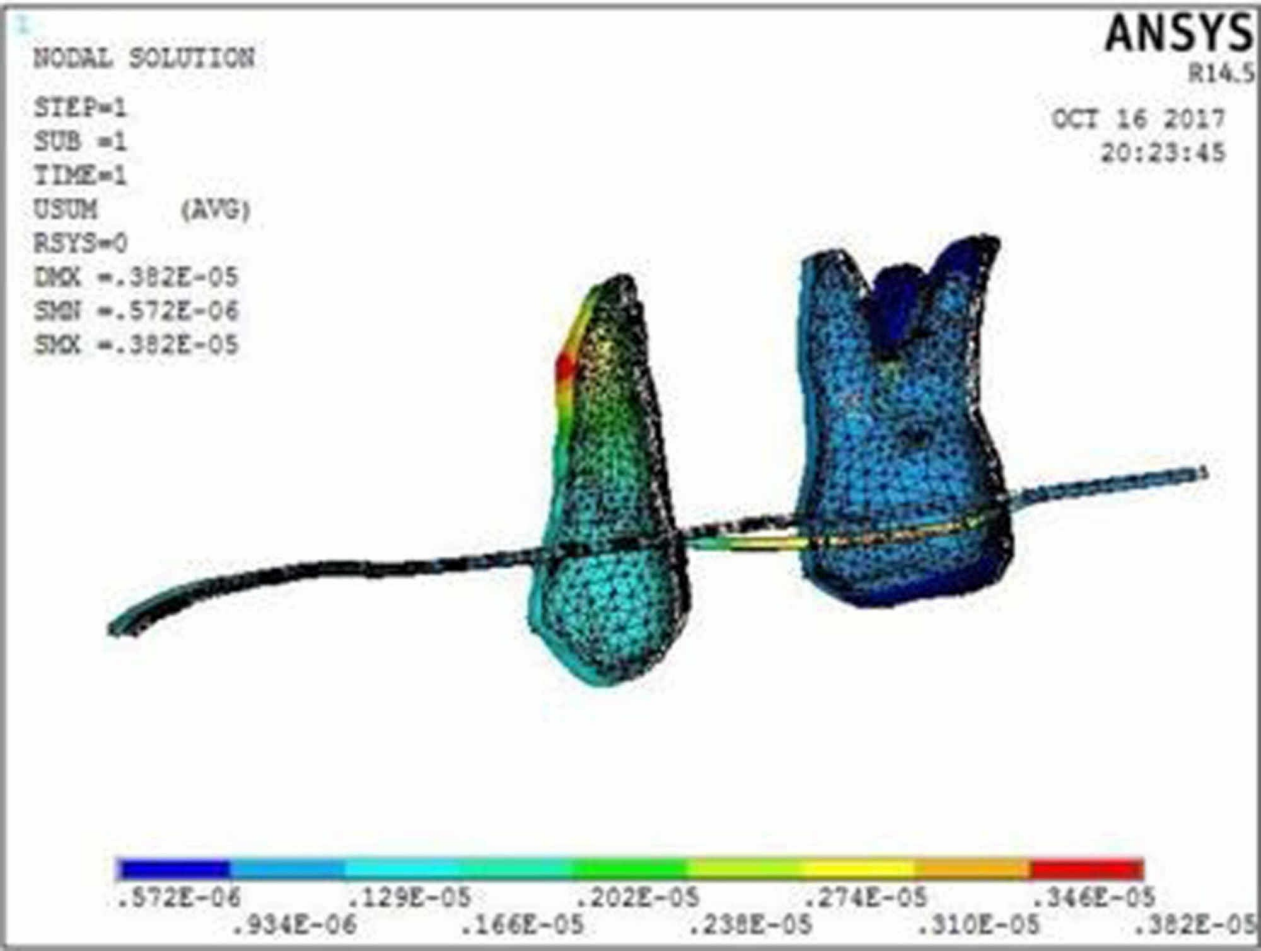
Deformation in the arch wire at the power arm height of 6 mm

**Figure 7:**
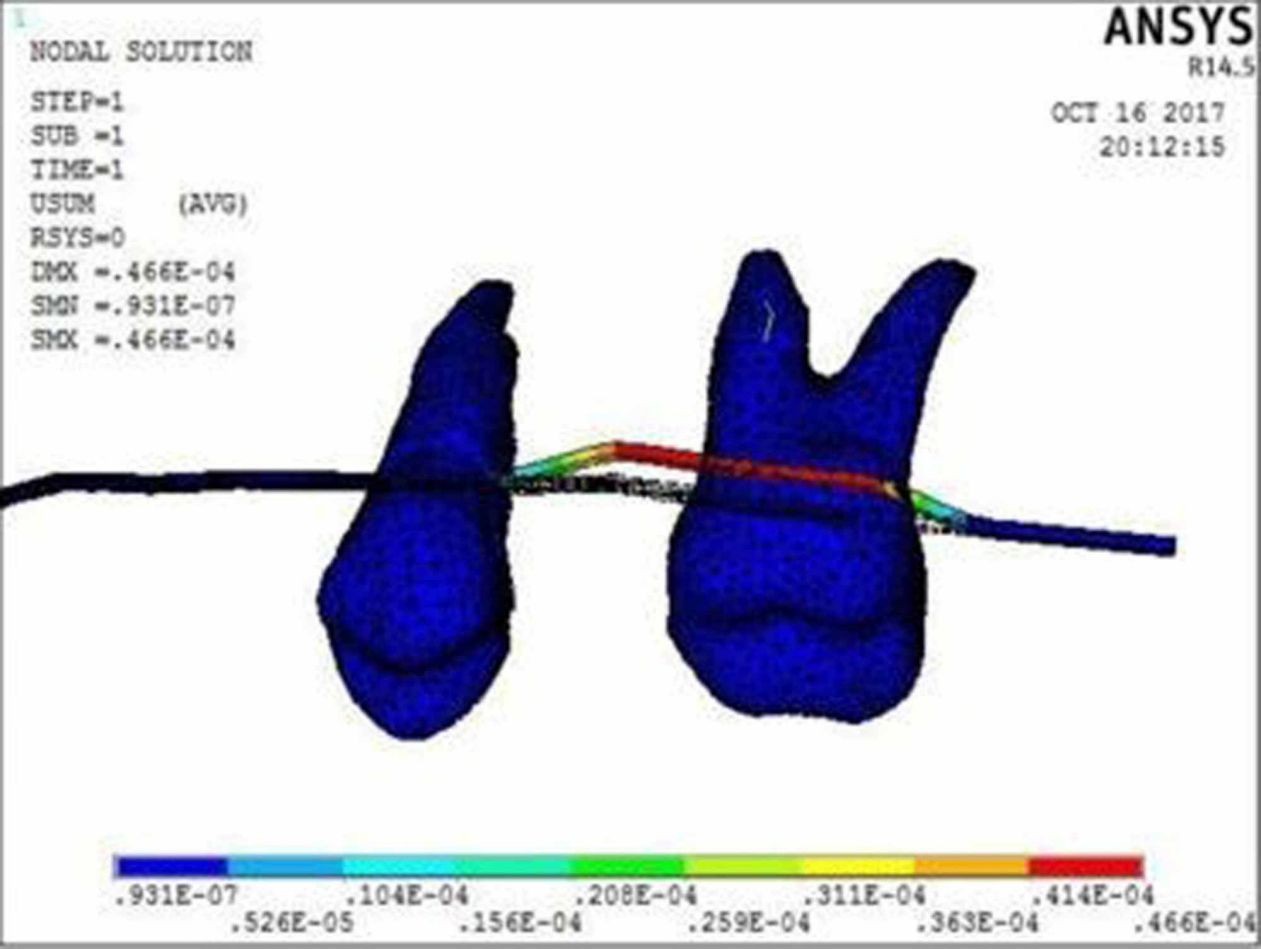
Deformation in the archwire at the power arm height of 10 mm

The archwire deformation at the heights of 2 mm, 6 mm, and 10 mm is illustrated in Figures 5-7, respectively.

 

When 0.017” × 0.022” archwire is engaged into the 0.018” slot bracket, and a force was applied at power arm of 2 mm, more tipping at the crown (8.75 µmm) than root (1.91 µmm) of the maxillary molar was observed. As the height of the power arm was raised apically, at 6 mm and 10 mm, the direction of tooth movement was more controlled. At the level of 10 mm, the difference between crown (0.09 µmm) and root (0.16 µmm) tipping was significantly less (0.07 µmm), indicating more bodily movement.

The archwire deflection is downward at when the power arm height is 2 mm and 6 mm, and the deflection is upward at 10-mm height in model 1. The arch wire deflection is downward at 2 mm and upward at 6 mm and 10 mm in model 2.

In model 2, at 2 mm, mesial crown tipping (8.56 µmm) was more than root movement (1.82 µmm). At 6 mm, there was more bodily movement with crown tipping of 1.75 µmm and root tipping of 1.26 µmm. At 10 mm, crown tipping of 0.521 µmm and root tipping of 0.24 µmm was noted.

**Figure 8:**
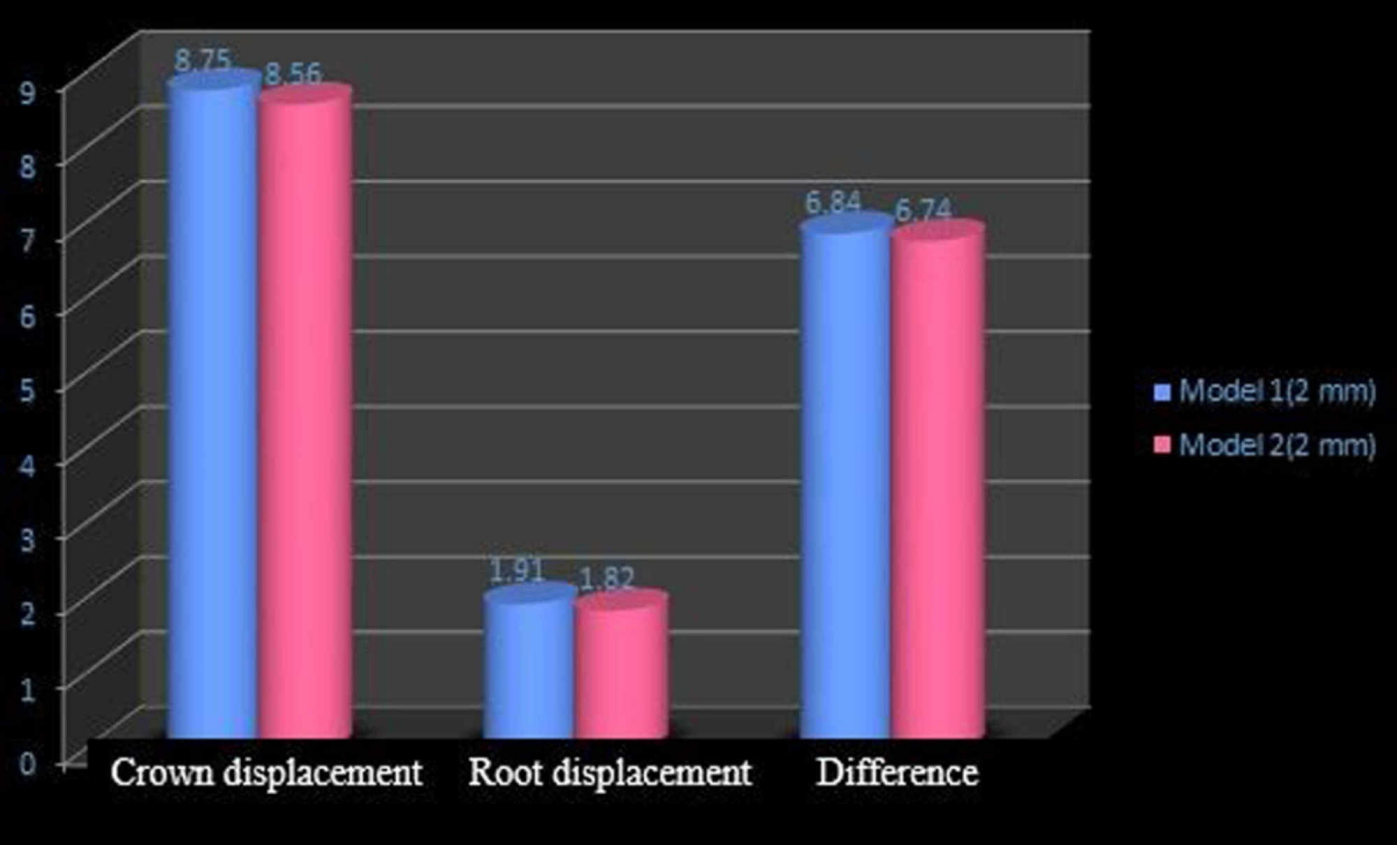
Comparison of molar displacement in models 1 and 2 at the power arm height of 2 mm

**Figure 9:**
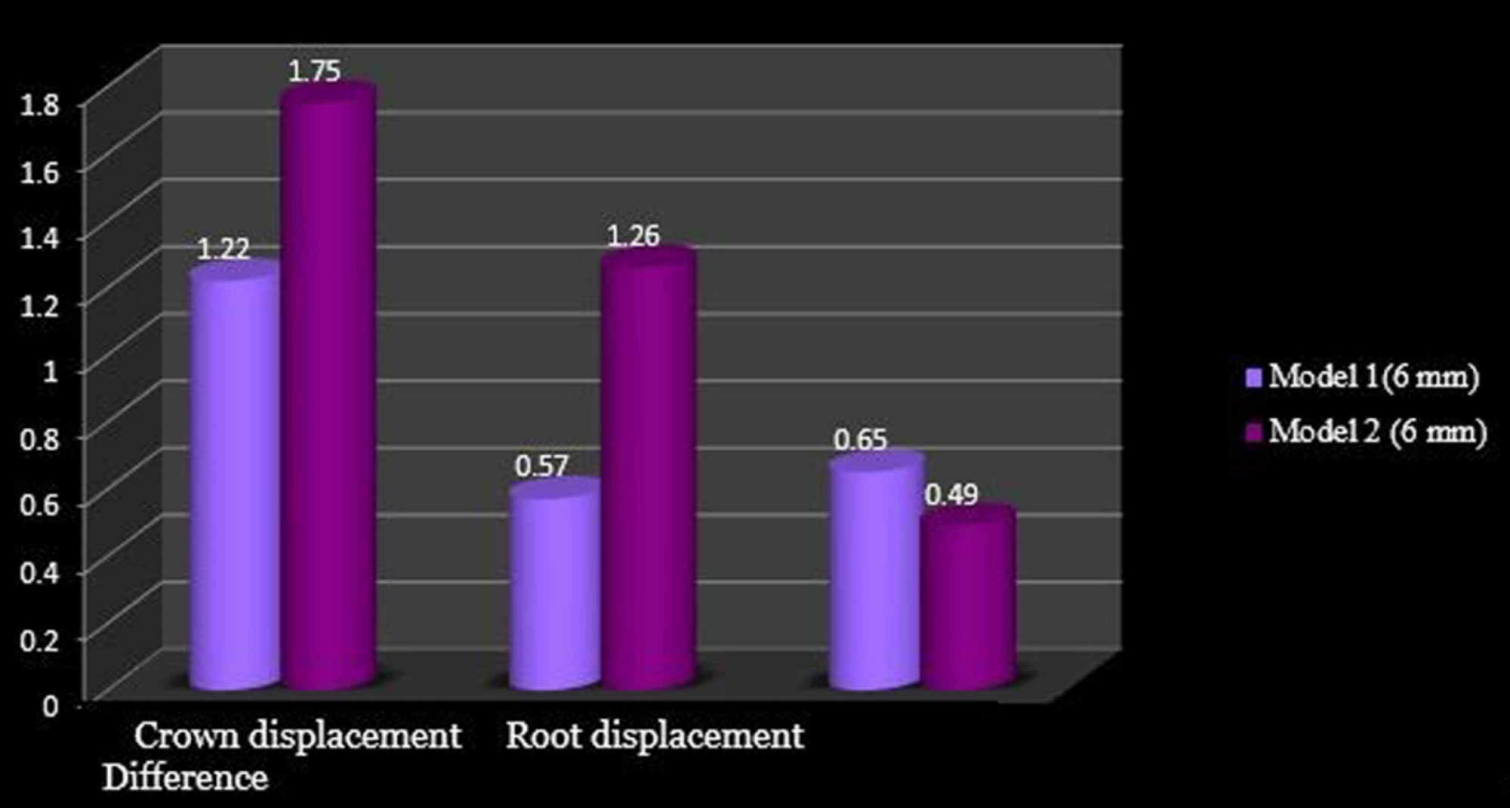
Comparison of molar displacement at the power arm height of 6 mm

**Figure 10:**
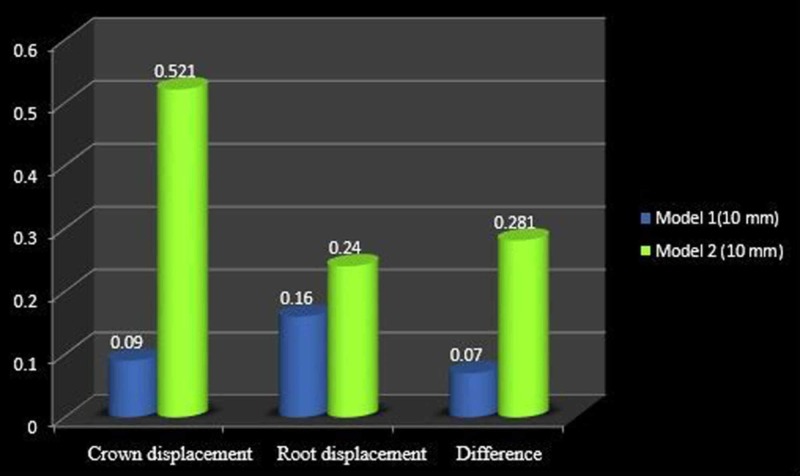
Comparison of molar displacement at the power arm height of 10 mm

The displacement of the tooth as a whole was more in model 1 compared to that in model 2 although more root movement was noted which indicates uncontrolled mesial crown tipping. In both cases, the deformation of the archwire was upwards. The comparison of the crown and root movement of model 1 and model 2 at the height of 2 mm, 6 mm, and 10 mm is shown in Figures 8-10, respectively. 

## Discussion

Miniscrews have been used as an anchor unit in challenging clinical situations such as space closure by maxillary molar protraction to avoid unwanted tooth movement [6]. Bracket slot dimensions, archwire size, stiffness, and the CR are important factors to be considered to avoid uncontrolled tipping of tooth. When force was applied through the CR, bodily tooth movement was seen. A few studies have shown that the CR of multi-rooted tooth lies 1-2 mm apically from the furcation. An FEM study carried out on mandibular molar protraction showed that there was least mesio-distal tipping, wherein force was applied at the height of 6 mm and 8 mm, which approximates the location of the predicted CR [7].

In this study, when 0.017” × 0.022” stainless steel archwire was engaged into 0.018” slot brackets, and a protraction force was applied at 2-mm height of the power arm, crown movement (8.75 µmm) was observed to be greater than root movement (1.91 µmm) of the maxillary molar, indicating uncontrolled crown tipping with the archwire deflecting downwards. As the height of the power arm was raised apically, at 6 mm and 10 mm, the direction of tooth movement changed to a more controlled crown tipping. At the level of 6 mm, however, the crown movement was 1.22 µmm and root movement was 0.57 µmm, with archwire deflecting downwards. At 10 mm, the root movement (0.16 µmm) was greater than the crown movement (0.09 µmm), with archwire deflecting upward.

In the 0.019” × 0.025” stainless steel archwire in the 0.022” slot bracket model, at the height of 2 mm, mesial crown movement (8.56 µmm) was greater than the root movement (1.82 µmm), with the archwire deflecting downward. At the 6-mm height of the power arm, there was crown movement of 1.75 µmm and a comparable root movement of 1.26 µmm and archwire deflecting upward. At a height of 10 mm, the protraction force produced a crown movement of 0.52 µmm and root movement of 0.24 µmm, with the archwire deflecting upward.

At the 6-mm height, model 2 showed more controlled mesial crown tipping. At the 10-mm height, model 1 showed controlled tooth movement compared to model two [8].

Less mesial root movement was produced with 0.019” × 0.025” archwire in the 0.022” slot, which has a larger play. The vertical dimension of the play in the 0.022” slot is three times as large as that in the 0.018” slot, but a horizontal dimension of the play is same. This indicates that play in the vertical dimension has a greater impact on tooth movement than in the horizontal dimension [9]. It is considered that lesser the play between the archwire and the bracket, greater are the forces. As a result, more mesial root tipping moment is transmitted to the molar in the 0.018-in slot system. The dimension of the play between the bracket slot and the archwire has a significant impact on posterior tooth movement when the protraction force was applied to a power arm. As the flexural rigidity of the archwire is determined by its material and cross-sectional shape, even if the same height of the horizontal protraction forces was applied, there were great discrepancies in the types of posterior tooth movement between the two combinations of bracket slot size and archwire dimension [8]. As the height of the forces applied increased, this discrepancy decreased.

At the 6-mm height of the power arm in models 1 and 2, the effect of play in the archwire is comparable (Figure 9), whereas as the height of the protraction force is raised apically, the play is more evident with a greater difference in the crown and root movement (Figure 10).

Limitations of the study: Only two archwire bracket slot combinations were tested and the effects of change in the position of the miniscrew were not considered. Certain assumptions made to simulate the physical environment may result in errors. We used ANSYS 11.0, an older version; currently, newer versions are available, which may give better FEMs.

## Conclusions

The current study indicates that in model 1, a power arm height of 10 mm provided controlled tooth movement compared to the one of 6 mm. In model two, power arms of both 6-mm and 10-mm height produced controlled tooth movement. However, a power arm of height 6 mm is more feasible than the one of 10 mm for effective molar protraction using 0.017” × 0.025” and 0.019”× 0.025” archwires in 0.018” and 0.022” slot, respectively, due to the anatomic limitations of vestibular height.

It was concluded that the degree of play as determined by the clearance of the archwire size in the bracket slot has a great impact on the control of posterior teeth movement. The height of the power arm must be closer to the center of resistance of the posterior teeth to obtain controlled tooth movement during space closure.
